# Breadth, phenotype and functionality of Gag-specific T cell responses induced by a heterologous DNA/MVA prime-boost HIV-1 vaccine regimen

**DOI:** 10.1186/1742-4690-9-S2-P273

**Published:** 2012-09-13

**Authors:** L Podola, A Bauer, A Haule, L Sudi, C Nilsson, K Godoy-Ramirez, P Mann, M Missanga, B Kaluwa, L Maboko, C Lueer, M Mwakatima, S Aboud, M Bakari, J Currier, M Robb, S McCormack, S Joseph, E Lyamuya, M Hoelscher, B Wahren, E Sandström, G Biberfeld, C Geldmacher, A Kroidl

**Affiliations:** 1 Ludwig-Maximilians University of Munich, Mbeya, Tanzania, United Republic of; 2 Mbeya Medical Research Programme, Mbeya, Tanzania, United Republic of; 3 Karolinska Institute and Swedish Institute for Communicable Disease, Solna, Sweden; 4 Muhimbili University of Health and Allied Sciences (MUHAS), Dar Es Salaam, Tanzania, United Republic of; 5 US Military HIV Research Program, Rockville, MD, USA; 6 MRC Clinical Trials Unit, London, UK

## Background

Broad Gag recognition and polyfunctionality of vaccine-induced AIDS virus-specific T cell responses correlate with better viral control in non-human primates and also in chronically HIV-infected individuals.

## Methods

Breadth and polyfunctionality of HIV-vaccine induced Gag-specific T cell responses were investigated in healthy Tanzanian volunteers who participated in the Tanzania Mozambique HIV vaccine trial (TaMoVac 01). Vaccine recipients received 3x 0.6 or 1.0mg intradermal injections of multiclade, multigene HIV-DNA vaccine boosted with 2x heterologous Modified Vaccinia Ankara (MVA)-CMDR. Using fresh peripheral blood mononuclear cells, the breadth of response was determined after the first MVA-CMDR boost using peptide pools for 9 successive Gag regions with an IFN-gamma ELISpot assay. Functionality (IFN-gamma, IL-2, TNF-alpha, Mip-1beta and the degranulation marker CD107) and phenotype (CD3, CD4, CD8) of HIV-specific T cells were assessed using flow cytometry in 52 participants after stimulation with peptide pools covering whole Gag-CMDR protein.

## Results

Two weeks after the first MVA-CMDR boost, a median of 2 Gag regions were recognized (range: 0-9, Placebos not excluded) by 45 participants. There was a strong linear correlation between the magnitude and the breadth of vaccine-induced Gag recognition (p<0.0001, r2=0.44). 13 (25%), 13 (25%), 3 (6%) and 29 (56%) of 52 subjects mounted IFN-gamma+ Gag-specific T cells that were either only CD4+, CD4+ & CD8+, only CD8+, or CD4+ and/or CD8+, respectively. Fifty percent of IFN-gamma+ Gag-specific CD4 T cells co-expressed TNF-alpha and/or IL-2. Co-expression of Mip-1beta or CD107 was reduced compared to CMVpp65-specific CD4 T-cells, which were measured simultaneously. More than 50% of IFN-gamma+ Gag-specific CD8 T-cells co-expressed CCR5 ligand Mip-1beta and a large proportion of these had degranulated.

## Conclusion

The TaMoVac-01 HIV-1 vaccine regimen induces a relatively broad Gag-specific response frequently dominated by CD4 T cells, many of which co-express IL-2 and/or TNF-alpha, but also induces detectable Gag-specific CD8 T-cells in a third of vaccine recipients.

